# Loss of erythrocyte arginase-1 impairs vasorelaxation due to endothelial GSNOR overexpression and denitrosylation of G protein subunits

**DOI:** 10.1016/j.redox.2026.104201

**Published:** 2026-05-04

**Authors:** Rajinikanth Gogiraju, Beichen Sun, Magdalena L. Bochenek, Ilka Wittig, Flávia Rezende, Melina Lopez, Angela Wirth, Wenjia Zhao, Elsa W. Böhm, Payal Guliani, Kateryna Moiko, Michael Molitor, Philip Wenzel, Marc Freichel, Philipp Lurz, Ralf P. Brandes, Katrin Schäfer

**Affiliations:** a Department of Cardiology, University Medical Center Mainz, Germany; b Deutsches Zentrum für Herz- und Kreislaufforschung (DZHK), Germany; c Center for Thrombosis and Hemostasis, University Medical Center Mainz, Germany; d Institute for Cardiovascular Physiology, Goethe University Frankfurt, Frankfurt am Main, Germany; e Institute of Pharmacology, University Medical Center Heidelberg, Germany; f Department of Ophthalmology, University Medical Center Mainz, Germany

**Keywords:** Arginase-1, Erythrocytes, Protein S nitrosylation, Vascular function

## Abstract

**Introduction:**

Overexpression of arginase-1 (ARG1) in red blood cells (RBCs) is associated with endothelial dysfunction, and short-term ARG1 inhibition or l-arginine supplementation restored endothelial function. However, the long-term consequences of ARG1 loss in RBCs and its effects on endothelial cells (ECs) are largely unknown. Here, we determined how deletion of ARG1 in RBCs affects blood pressure and vasorelaxation and the role of endothelial NO signaling via the soluble guanylyl cyclase and the S nitrosylation pathway for vascular homeostasis.

**Methods:**

Vascular function was monitored and vasorelaxation studied in C57BL/6 wild-type (WT) and apolipoprotein E deficient (apoE^−/−^) mice lacking ARG1 in cells of the erythrocyte lineage (RBC.ARG1-knockout, KO) and in mice with inducible deletion of ARG1 in ECs (END.ARG1-KO). Primary cells were analyzed using redox proteomics, immunolabeling and fluorescence microscopy.

**Results:**

Loss of ARG1 in RBCs resulted in significantly elevated plasma nitrite and lower mean and diastolic blood pressure levels, and increased phosphorylation of Vasodilator-Stimulated Phosphoprotein in smooth muscle cells indicated overactivated cyclic GMP signaling. In ECs, nitrosoglutathione reductase (GSNOR) overexpression and denitrosylation of endothelial Guanine Nucleotide Binding Proteins was observed, which may have uncoupled NO signaling from cGMP-mediated vasorelaxation in response to acetylcholine. Importantly, inhibition of GSNOR restored the impaired endothelium-dependent vasorelaxation in RBC.ARG1-KO aortas to levels comparable to RBC.ARG1-WT controls.

**Conclusions:**

Our data support the contribution of RBC-derived NO to blood pressure regulation, but also show that chronically elevated circulating NO levels induce counterregulatory mechanisms in ECs, including increased GSNOR expression and protein S-denitrosylation leading to impaired vasorelaxation despite overactivated cGMP signaling.

## Highlights

• RBC ARG1 deletion elevates circulating NO.
• Chronic NO exposure induces endothelial dysfunction.
• GSNOR inhibition restored vasorelaxation.

## Graphical abstract

Created in https://BioRender.com.

**Figure:**
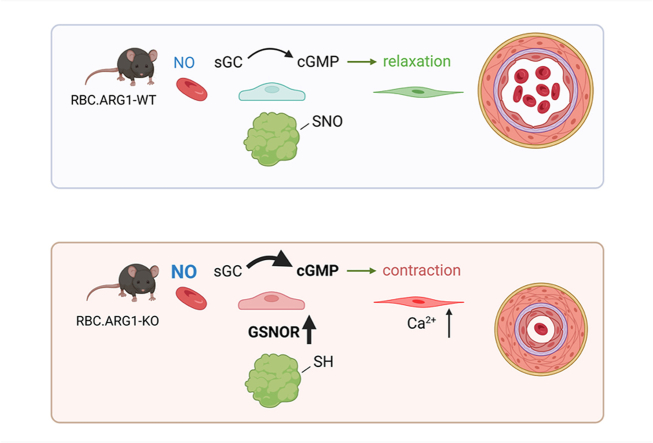

## 1 Introduction

Vascular relaxation is controlled by vasoactive factors produced in endothelial cells (ECs), and the inability of the endothelium to serve this function that is, endothelial dysfunction, is the cause of many cardiovascular disease entities [1]. Nitric oxide (NO), a critical mediator of vasorelaxation, is generated from endothelial NO synthase (eNOS), an enzyme expressed in ECs, but also in erythrocytes (red blood cells; RBCs) [2]. Studies in genetically engineered mice have shown that endothelial and erythrocyte eNOS both significantly contribute to systemic nitrite levels and blood pressure homeostasis, whereas vascular function is mainly controlled by NO derived from endothelial, not erythrocyte eNOS [3,4]. The biosynthesis of NO in the vascular system is counterregulated by arginase, which competes with eNOS for their common substrate l-arginine [5,6]. Like eNOS, the type 1 isoform of arginase (ARG1) is expressed in ECs and RBCs [5,7]. Increased expression of ARG1 was reported in erythrocytes from patients with metabolic cardiovascular risk factors and linked with vascular dysfunction, and ARG1 inhibition or l-arginine supplementation restored endothelial function [[8], [9], [10]]. However, whether the ARG1 overexpression seen in patient RBCs led to alterations in cGMP-dependent and -independent NO signaling in vascular cells was never studied.

## 2 Methods

### 2.1 Experimental animals

We generated two conditional mouse lines lacking ARG1 in either erythrocytes (RBC.ARG1-KO) or ECs (END.ARG1-KO) by crossing transgenic mice expressing *loxP*-flanked (floxed) exon 7 and 8 of the Arg*1* gene (ARG1^fl/fl^; RRID:IMSR_JAX:008817) with mice expressing Cre recombinase under control of either the constitutively active erythropoietin receptor promoter (EpoR; courtesy of Professor Ursula Klingmüller, DKFZ, Heidelberg, Germany [11]) or the tamoxifen-inducible TEK receptor tyrosine kinase promoter (Tie2.ER^T2^; courtesy of Professor Bernd Arnold, DKFZ, Heidelberg, Germany [12]). To examine the importance of erythrocyte ARG1 in the context of hypercholesterolemia, RBC.ARG1-KO mice were backcrossed onto the apolipoprotein E deficient (apoE^−/−^) background [13]. All animal experiments had been á priori approved by the local animal ethics committee and the *Landesuntersuchungsamt Rheinland-Pfalz* (animal permits G16-1-003 and G22-1-044) and complied with national guidelines for the care and use of laboratory animals and the ARRIVE guidelines [14].

### 2.2 Primary mouse erythrocyte isolation and analysis

Erythrocytes were isolated from 3.8% sodium citrate-anticoagulated whole blood of anesthetized RBC.ARG1-WT and RBC.ARG1-KO mice, as described [13]. For immunostaining and confocal microscopy, erythrocytes were fixed in 0.5% glutaraldehyde (Carl Roth) in 1X phosphate-buffered saline (PBS; Gibco) for 5 min at room temperature (RT). After fixation, erythrocytes were washed three times in rinsing buffer (0.1 M glycine in PBS) to block unreacted aldehydes and then permeabilized using 0.1% Triton X-100 (Carl Roth) for 5 min. Erythrocytes were incubated for 60 min in blocking buffer (0.05 mM glycine, 0.2% fish skin gelatin and 0.05% sodium azide in PBS) followed by incubation with monoclonal antibodies against ARG1 (Cell Signaling Technology Cat# 93668, RRID:AB_2800207). Erythrocyte membranes were visualized using Wheat Germ Agglutinin (WGA; Alexa Fluor™ 488 conjugate; Invitrogen; catalogue number W11261). Staining results were observed on a confocal microscope (Leica TCS SP8) under a 60X oil immersion objective lens and evaluated using Leica Application Suite X software (LAS X).

Plasma nitrite levels were determined using chemiluminescence technology (Sievers 280i Nitric Oxide Analyzer), as described previously [15]. In brief, mice received bottled water with low nitrite levels (evian® natural spring water) for at least one week prior to measurements to minimize the detection of background nitrite levels. Whole blood was collected in heparinized tubes (Microvette® 300 LH, Ref 20.1309, SARSTEDT AG &Co. KG) and centrifuged at 1500 g for 15 min at 4 °C to collect plasma. Plasma was further processed using centrifugal filter units (Microcon®-10, Merck #MRCPRT010) by centrifugation at 17,000 g for 60 min at 4 °C. Subsequently, 50 μL of the deproteinized plasma was injected into nitric oxide analyser to measure plasma nitrite levels.

### 2.3 Primary mouse endothelial cell isolation and analysis

Primary ECs were isolated from murine lungs using platelet endothelial cell adhesion molecule (CD31)-conjugated magnetic MicroBeads (catalogue number 130-097-418) and sorted using magnetic separation LS columns (catalogue number 130-042-401) (both from Miltenyi Biotec), as described in more detail elsewhere [16], and seeded on 10 cm plates coated with 0.2% gelatin (Sigma-Aldrich; catalogue number G1393). Using this methodology, lung ECs can be isolated from mice with high yield and purity [17,18]. Cells were expanded by cultivation in Endothelial Cell Growth Medium MV2 Kit (PromoCell; C-22121) for 4 days, followed by preparation for immunocytochemical analysis, as described [16], using primary antibodies to detect differences in GSNOR (Proteintech catalogue number CL488-16379, RRID: AB_2919133) expression. Primary smooth muscle cells were isolated from the murine aorta, as described [19], and expanded in Dulbecco's Modified Eagles Medium (DMEM; Gibco; catalogue number 31966-021) until subconfluency followed by preparation for cytosolic Ca^2+^ measurements or immunoblot analysis.

### 2.4 Invasive blood pressure measurements

Telemetric blood pressure measurements were performed, as described previously [20]. Briefly, PA-C10 implants (Data Sciences International) were used, and data were collected with Ponemah acquisition software (version 6.40). Blood pressure data were collected with a 10-s scheduled sampling every 5 min, and the 24-h mean values were used for analysis. l-NAME (Sigma-Aldrich; catalogue number N5751) was administered at a dosage of 1 mg/mL via the drinking water. NorNOHA (10 mg/kg; Cayman; catalogue number 10006861) was administered intraperitonally as a bolus once daily. Blood pressure data for each substance were recorded over a period of 3 days. A break of at least 3 days was kept in-between the treatments. The animal procedures for the invasive blood pressure analysis had been á priori approved from the local authorities (Regierungspräsidium Karlsruhe, 35-9185.81/G-202/20, addendum approved 05.03.2025).

### 2.5 Vascular relaxation studies in isolated thoracic aortic ring segments

The thoracic aorta was carefully prepared from deeply anesthetized mice and cut into two 3 mm-long segments. The aortic rings were mounted on force transducers in organ chambers and first preconstricted with prostaglandin F2α (3 nM) to reach 50 to 80% of the tone induced by KCl, as described [21]. Immediately afterwards, aortic ring segments were subjected to vascular relaxation studies in response to increasing dosages of acetylcholine (ACh; from 10^−9^ to 10^−5.5^ M, Sigma-Aldrich, catalogue number A6625) or glyceryl trinitrate (GTN; from 10^−9^ to 10^−4.5^ M, Pohl-Boskamp, catalogue number PZN16511168). To inhibit GSNOR, aortic rings from C57BL/6 wild-type mice were incubated overnight, following a protocol reported by others [8,22], in the presence or absence of N6022 (100 nM, previously established as effective in inhibiting GSNOR [13,23]; Cayman Chemicals, catalogue number 21269) along with intact RBCs isolated from RBC.ARG1-WT and RBC.ARG1-KO mice and diluted to 20% hematocrit in DMEM. Equal volumes of DMSO were used as vehicle control.

### 2.6 Microvascular function studies in mesenteric arteries

Microvascular function was analyzed by pressure myography and videomicroscopy, as previously described [24]. In brief, deeply anesthetized mice were killed by cervical translocation, and the mesenteric vascular bed was excised and immediately transferred to ice-cold Krebs buffer. Resistance mesenteric arteries were carefully isolated and cleaned from the surrounding tissue under a dissection microscope. The isolated vessel was then transferred to an organ chamber, cannulated at both ends to the tips of a glass micropipettes and secured using 10.0 nylon sutures. The organ chamber was continuously circulated with oxygenated Krebs buffer maintained at 37 °C using a peristaltic pump, and the vessel was pressurized to 50 mmHg under no-flow conditions. The mesenteric arterioles were visualized using an inverted light microscope and changes in luminal diameter were recorded and measured using an on-screen ruler. After confirming the vessel viability by depolarization-induced constriction with KCl (100 mM), resulting in at least a 30% reduction to the initial diameter, concentration response curves for the thromboxane A_2_ analog U46619 (10^−11^ to 10^−6^ M; Cayman Chemical, catalogue number 56985-40-1) were generated to induce vascular tone. Vasodilatory responses were assessed by cumulative addition of the endothelium-dependent agonist acetylcholine (10^−9^ to 10^−4^ M; Sigma-Aldrich, catalogue number A6625) or the endothelium-independent nitric oxide donor sodium nitroprusside (10^−9^ to 10^−4^ M; Sigma Aldrich, catalogue number 567538).

### 2.7 Primary mouse smooth muscle cell isolation and analysis

Primary SMCs were isolated from the aorta of RBC.ARG1 WT and RBC.ARG1-KO mice, cut into small pieces and digested in DMEM containing collagenase II (5 mg/mL; Gibco). Medium was removed by centrifugation, and cells were resuspended in DMEM containing 10% fetal bovine serum (FBS; Gibco), 100 U/mL penicillin and 100 μg/mL streptomycin (Gibco), and seeded onto 0.2% gelatin-coated 6-well plates. Cells were cultivated in a humidified 5% CO_2_ atmosphere at 37 °C.

Cytosolic calcium levels in primary aortic smooth muscle cells were assessed using the fluorescence-based calcium assay kit Fluo-4 Direct™ Calcium Assay Kit (Invitrogen, catalogue number F10471; excitation 494 nm, emission 516 nm). Time-lapse images were captured before and after the addition of 10^−5.5^ M acetylcholine. Image analysis was performed using ImageJ (NIH, USA), where the mean fluorescence intensity (MFI) of individual cells was measured at each time point to quantify the calcium response. Results were normalized to the baseline fluorescence and reflect relative changes in cytosolic calcium levels in response to ACh stimulation. Equal numbers of cells were analyzed to ensure consistency and comparability.

### 2.8 Immunostaining and fluorescence microscopy

Acetone-fixed cross-sections through the aorta and the mesenteric artery of apoE^−/−^ RBC.ARG1-WT and apoE^−/−^ RBC.ARG1-KO mice were incubated with antibodies against GSNOR (Proteintech Cat# CL488-16379, RRID: AB_2919133). Cell nuclei were stained using 4′ 6-diamidino-2-phenylindole (DAPI). ECs were visualized using monoclonal antibodies against PECAM1 (Santa Cruz Biotechnology Cat# sc-18916, RRID: AB_627028). Images were taken using a Keyence BZ-X800E microscope and analyzed using BZ-X800 analysis software (Keyence Corporation).

### 2.9 Western blot analysis

At 80-90% confluency, primary murine ECs or SMCs were lysed in ice-cold RIPA buffer (Cell Signaling Technology; catalogue number 9806) containing 1 mM phenylmethylsulfonyl fluoride (PMSF; Cell Signaling Technology; catalogue number 8553) and protease inhibitor cocktail (Thermo Scientific; catalogue number 78444). Protein lysates (50 μg) were separated using SDS polyacrylamide gel electrophoresis with molecular weight standards (BioRad; catalogue number 1610374) and transferred to nitrocellulose membranes (Protran, Whatman; catalogue number 11925104). Electrophoresis was performed on 10% gels (self-prepared) or 4-15% gradient precast polyacrylamide gels (BioRad; catalogue number 4561083). Subsequently, the membranes were blocked in 5% BSA in tris-buffered saline (TBS) containing 0.1% Tween-20 for 1 h at RT and incubated overnight at 4 °C with primary antibodies against murine ARG1 (Cell Signaling Technology catalogue number 93668, RRID:AB_2800207), GSNOR (Proteintech catalogue number 66193-1-Ig RRID:AB_2881587) or Phospho-VASP (Ser239) (Cell Signaling Technology catalogue number 3114, RRID:AB_2213396). Antibodies against β-actin (Abcam catalogue number ab20272, RRID:AB_445482) or actinin (Cell Signaling Technology catalogue number 3134, RRID:AB_2223798) were used to control for equal protein loading. Protein bands were visualized using horseradish peroxidase-conjugated secondary antibodies (GE Healthcare; catalogue numbers NA934 and NA931) and detected with Super Signal Chemiluminescent Substrate (Thermo Scientific; catalogue number 34579) on a VILBER Lourmat Fusion FX system, using the Fusion software. Densitometry analysis was performed using AlphaEaseFC 4.0 software.

### 2.10 Detection of protein S-nitrosylation

S-nitrosylated proteins in primary ECs isolated from lungs of RBC.ARG1-WT and RBC.ARG1-KO mice were detected using the Pierce™ S-Nitrosylation Western Blot kit (Thermo Scientific; catalogue number 90105). After isolation, primary ECs were immediately lysed by sonication in HENS buffer (consisting of 100 mM HEPES (pH 7.8), 1 mM EDTA, 0.1 mM neocuproine, and 1% sodium dodecyl sulfate [SDS]) and centrifuged at 10,000 g for 10 min to reduce viscosity. The protein concentration was determined using BCA reagent (Thermo Scientific). Equal amount of protein (100 μg) in 100 μL volume was taken per sample to process for iodo TMT assay. 2 μL of 1 M MMTS (20 mM final concentration) was added to each sample, mixed by briefly vortexing it, and incubated for 30 min at RT to block the free cysteine thiols. Then, 600 μL (six volumes) of pre-chilled (−20 °C) acetone was added and incubated for at least 1 h at −20 °C to precipitate MMTS followed by centrifugation (at 12,000×*g*) for 10 min at 4 °C. The tubes were carefully inverted to decant the acetone, and the pellet was air-dried for 15 min. The dried pellet was re-suspended in 100 μL of HENS buffer and 1 μl of the iodoTMT labeling reagent was added followed by the 2 μL of 1 M sodium ascorbate was added and briefly vortexed to mix and incubated at room temperature for 2 h. Unbound iodoTMT was removed by another round of precipitation step with acetone. The final pellet was resuspended in 30 μL HENS buffer, and the samples were subjected to immunoblotting using anti-TMT antibody (dilution, 1:1000) provided along with the kit. Nitrosylated protein signals, detected using the anti-TMT antibody, were normalized first to the corresponding ACTB band intensity for each lane, and these values were then further normalized to the mean of the WT group to compare the relative changes between the genotypes.

### 2.11 Identification of S-nitrosylated proteins using the BIAM switch assay

S-nitrosylated proteins were detected using the BIAM (biotinylated iodoacetamide) switch assay follwed by mass spectrometry, essentially as described [25], except that ascorbic acid/copper was used as the reducing reagent. The complete mass spectrometry proteomics dataset, including raw and processed data, metadata, and a detailed method description, has been deposited with the ProteomeXchange Consortium via the PRIDE partner repository (http://proteomecentral.proteomexchange.org) under the dataset identifier PXD075167 and 10.6019/PXD075167.

### 2.12 Statistical analysis

Data were analyzed for normal distribution using the Shapiro-Wilk test and Bartlett and Brown-Forsythe test for equal variance. If normal distribution was present, data are presented as mean ± standard error of the mean (SEM) and were compared using unpaired *t*-test (for two groups) or one-way ANOVA, Sidak's multiple comparisons test (for more than two groups). If normal distribution was not present, data are shown as median ± interquartile range and compared using Mann-Whitney test or Kruskall-Wallis, Dunn's multiple comparisons test. Two-way ANOVA was used to compare data in more than two groups and an additional variable (e.g. time). Statistical significance was assumed when P value was lower than 0.05. All analyses were performed using data analysis software (version 10.1.2; GraphPad Software).

## 3 Results

**Fig. 1:**
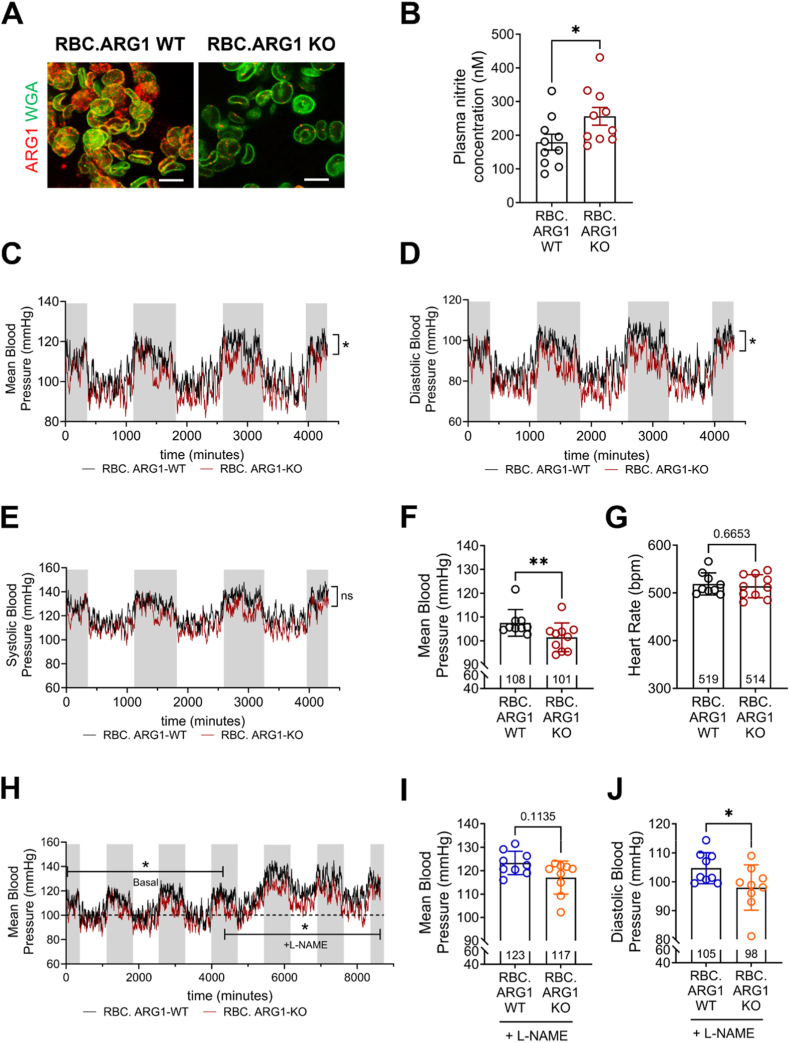
Loss of arginase 1 in erythrocytes elevates circulating nitrite levels and lowers mean and diastolic blood pressure levels. **A** Representative confocal microscopy images of ARG1 (red signal) in erythrocytes from C57BL/6 RBC.ARG1-WT or C57BL/6 RBC.ARG1-KO mice. Erythrocyte membranes were visualized using Wheat Germ Agglutinin (WGA, green signal). Size bars represent 5 μm **B** Plasma nitrite levels (determined using Sievers 280i Nitric Oxide Analyzer) in C57BL/6 RBC.ARG1-WT or C57BL/6 RBC.ARG1-KO (n = 10 biological replicates, representative of two independent experiments). *P < 0.05 (unpaired Student's *t*-test). **C-E** Longitudinal blood pressure changes of continuous three days radiotelemetry recordings of the mean (C), diastolic (D) and systolic (E) arterial blood pressure in C57BL/6 RBC.ARG1-WT (black; n = 9) and C57BL/6 RBC.ARG1-KO (red; n = 10) mice during alternating dark/light cycles. Mixed-effects analysis revealed significant effects of time (C-E; P < 0.0001; not shown) and genotype (*P < 0.05 in C and D; ns, non-significant in E). **F, G** Three day average values of mean blood pressure (F) and baseline heart rate (G) in C57BL/6 RBC.ARG1-WT (n = 9) and C57BL/6 RBC.ARG1-KO mice (n = 10). **P < 0.01 (Mann-Whitney test in F). **H** Comparison of continuous six day radiotelemetry recordings of mean blood pressure in C57BL/6 RBC.ARG1-WT (black) and C57BL/6 RBC.ARG1-KO (red) mice, untreated and in response to NOS inhibition using l-NAME (1 mg/mL in the drinking water for 3 days). Mixed-effects analysis showed significant effects of time for basal and l-NAME conditions (P < 0.0001 for both; not shown) and genotype for basal (*P = 0.0355) and l-NAME conditions (*P = 0.0209). No significant differences in time vs. genotype interaction in either condition, indicating similar temporal profiles between the groups. **I, J** Three day average values of mean blood pressure (I) and heart rate (J) in l-NAME treated C57BL/6 RBC.ARG1-WT (n = 9) and C57BL/6 RBC.ARG1-KO mice (n = 9). *P < 0.05 (Mann-Whitney test).

***Loss of ARG1 in erythrocytes elevates circulating nitrite levels and lowers mean and diastolic blood pressure levels.*** To determine the long-term consequences of interfering with the NO biosynthetic pathway in erythrocytes, we generated a transgenic mouse line with ARG1 deletion in the erythrocyte lineage starting at the early progenitor stage (Fig. 1A). We previously documented significantly reduced erythrocyte mRNA and protein ARG1 expression levels resulting in increased erythrocyte NO release and also ruled out off-target Cre recombinase mediated gene deletion in EpoR-Cre expressing non-erythroid cells [13]. Plasma nitrite levels were significantly elevated in the RBC.ARG1-KO mice compared to RBC.ARG1-WT controls (Fig. 1B), in line with reported contribution of RBC eNOS to the circulating NO pool [4]. Longitudinal invasive blood pressure measurements over three days showed significantly reduced diastolic and mean blood pressure levels in RBC.ARG1-KO compared to RBC.ARG1-WT mice, whereas systolic blood pressure was not altered (Fig. 1C–E). Although the mean difference (∼7 mmHg; Fig. 1F) may seem minor, clinical evidence has shown significant health benefits and cardiovascular risk reductions for lowering blood pressure at similar levels [26]. RBC.ARG1-WT and RBC.ARG1-KO mice both responded to systemic treatment with the NOS inhibitor l-NAME with an increase in mean blood pressure levels, whereas diastolic blood pressure levels continued to be significantly lower in l-NAME treated RBC.ARG1-KO compared to l-NAME-treated RBC.RG1-WT mice (Fig. 1H–J). Although the administration of l-NAME to mice via the drinking water unavoidably inhibits both erythrocyte and endothelial eNOS, these data are in line with increased l-arginine substrate utilization via eNOS towards increased NO biosynthesis.

**Fig. 2:**
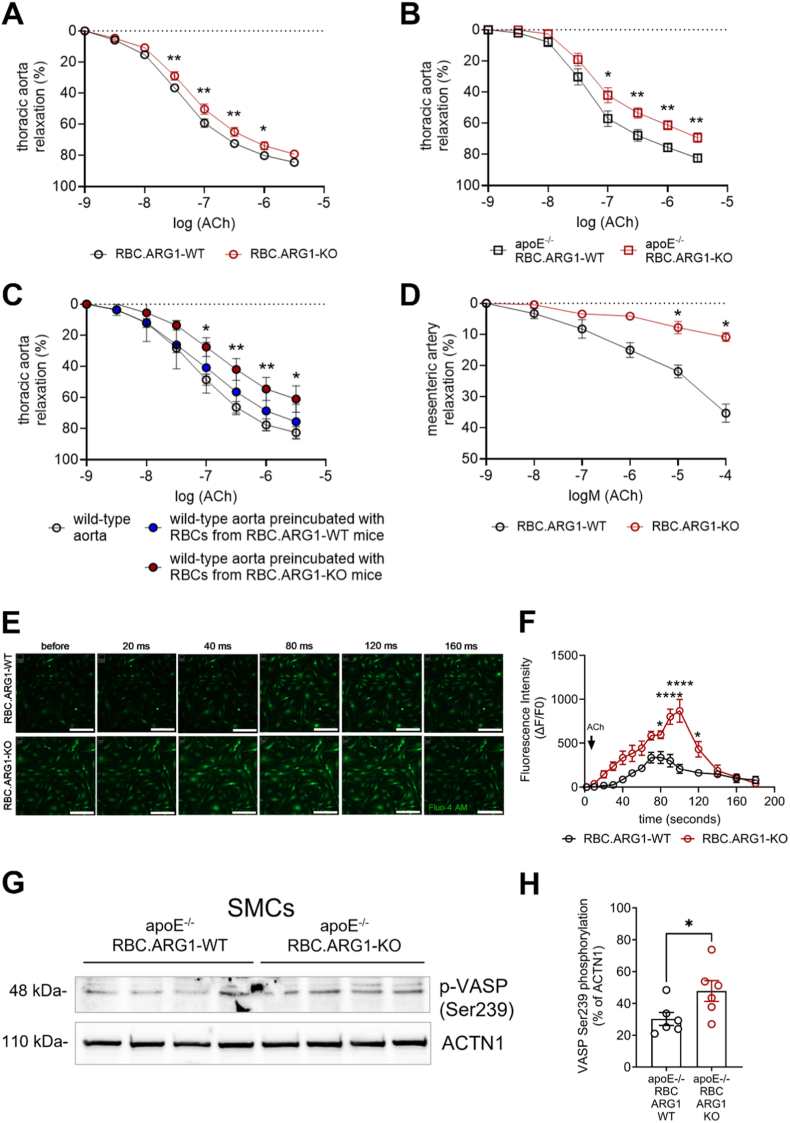
Mice lacking arginase-1 in erythrocytes respond to acetylcholine with impaired endothelium-dependent vasorelaxation and stronger cytosolic calcium increase in aortic smooth muscle cells **A** Concentration–relaxation curves in response to increasing dosages of the endothelium-dependent vasodilator acetylcholine (ACh; 10^−9^ to 10^−5.5^ M) in thoracic aorta segments isolated from C57BL/6 background mice. Summary of findings in n = 17C57BL/6 RBC.ARG1-WT and n = 20C57BL/6 RBC.ARG1-KO mice. **B** Concentration–relaxation curves in response to acetylcholine (10^−9^–10^−5.5^ M) in thoracic aortic segments isolated from apoE^−/−^ background mice. Summary of the data from apoE^−/−^ RBC.ARG1-WT (n = 6) and apoE^−/−^ RBC.ARG1-KO (n = 10) mice are shown. *P < 0.05 and **P < 0.01 vs. WT controls at the same ACh concentration (two-way ANOVA). **C** Vascular function was examined in thoracic aortic segments from C57BL/6 wild-type mice (n = 8), co-incubated overnight with erythrocytes (20% hematocrit) freshly isolated from RBC.ARG1-WT and RBC.ARG1-KO mice. *P < 0.05 and **P < 0.01 vs. WT aorta control (two-way ANOVA). **D** Concentration–relaxation curves in response to ACh (10^−9^ to 10^−4^ M) in mesenteric artery segments isolated from C57BL/6 background mice. Summary of findings in n = 3C57BL/6 RBC.ARG1-WT and n = 3C57BL/6 RBC.ARG1-KO mice. *P < 0.05 vs. WT controls at the same ACh concentration (two-way ANOVA). **E, F** Changes in intercellular Ca^2+^ concentrations in primary aortic smooth muscle cells isolated from C57BL/6 RBC.ARG1-WT or RBC.ARG1-KO mice (n = 3 biological replicates per genotype). Cells were incubated with the cell-permeable Ca^2+^ indicator Fluo-4 AM (green fluorescence) and examined immediately before and over 180 msec after stimulation with ACh (10^−5.5^ M). Representative images (E; size bars represent, 200 μm) and results of the quantitative analysis of calcium transients were quantified as ΔF/F_0_, where F_0_ represents the mean baseline fluorescence before stimulation (F). *P < 0.05 (two-way ANOVA). **G, H** Immunoblot analysis of VASP phosphorylated at Ser-239 in primary aortic smooth muscle cells (SMCs) isolated from apoE^−/−^ RBC.ARG1-WT and apoE^−/−^ RBC.ARG1-KO mice. ACTN1 was used as protein loading control. Representative membrane (G) and quantitative analysis (H). n = 6 biological replicates. *P < 0.0001 (unpaired Student's t-test).

***Mice lacking arginase-1 in erythrocytes respond to acetylcholine with impaired endothelium-dependent aortic vasorelaxation and stronger cytosolic calcium increase in aortic smooth muscle cells*.** To distinguish the contribution of endothelial versus erythrocyte derived NO for vascular NO signaling and function, we moved to the *ex vivo* situation and subjected thoracic aorta segments from RBC.ARG1-WT and RBC.ARG1-KO mice to vascular function studies. Unexpectedly, the eNOS-*in*dependent vasorelaxation of aortic rings from RBC.ARG1-KO mice to increasing concentrations of acetylcholine (ACh) was significantly impaired compared to those from RBC.ARG1-WT littermate controls examined in parallel (Fig. 2A). In contrast, vasodilation in response to the NO donor glyceryl trinitrate (GTN) did not differ (not shown). The vascular response to ACh was impaired to an even greater extent in RBC.ARG1-KO mice backcrossed onto the apoE^−/−^ background to induce hypercholesterolemia (Fig. 2B). Overnight incubation of wild-type mouse aortic segments with erythrocytes from RBC.ARG-KO, but not from RBC.ARG-WT mice was sufficient to significantly impair vasorelaxation in response to ACh (Fig. 2C), supporting a causal role of erythrocytes in our observations. To confirm this unexpected observation in the aorta, a vessel not primarily involved in blood pressure regulation, we also examined the response of mesenteric arterioles that is, prototype resistance vessels. While mesenteric arterioles from RBC.ARG1-WT mice responded to increasing ACh concentrations with vasodilation, the vasorelaxant response was significantly less pronounced in mesenteric arterioles from RBC.ARG1-KO littermate mice (Fig. 2D). Vascular relaxation of isolated thoracic aorta segments from mice lacking ARG1 in ECs (End.ARG1-KO) did not differ from End.ARG1-WT controls (not shown), corroborating earlier reports [27,28]. To understand the underlying mechanism, we examined primary vascular SMCs as the “main function performer regulating vasoconstriction and dilation” [29]. *Real-time* measurements of changes in cytosolic calcium (Ca^2+^) levels in response to the half-maximal effective ACh concentration revealed a significantly faster and more pronounced Ca^2+^ rise in aortic SMCs from RBC.ARG1-KO mice compared to RBC.ARG1-WT controls (Fig. 2E and F), consistent with the observed impaired vasorelaxation. Levels of vasodilatory-stimulated phosphoprotein (VASP), phosphorylated at the preferential protein kinase G activation site serine-239, were significantly upregulated in SMCs from RBC.ARG1-KO mice compared to RBC.ARG1-WT controls (Fig. 2G and H). Together, these findings suggest that chronic elevation of circulating nitrite levels *in vivo*, albeit in the nanomolar range, may have lead to overactivated NO−cyclic GMP signaling in SMCs from RBC.ARG1-KO mice and a pathological response to acetylcholine. Previous studies have shown that the sensitivity of soluble guanylyl cyclase to NO is in the nanomolar range [30] and sufficient to activate cGMP signaling in SMCs [31].

**Fig. 3:**
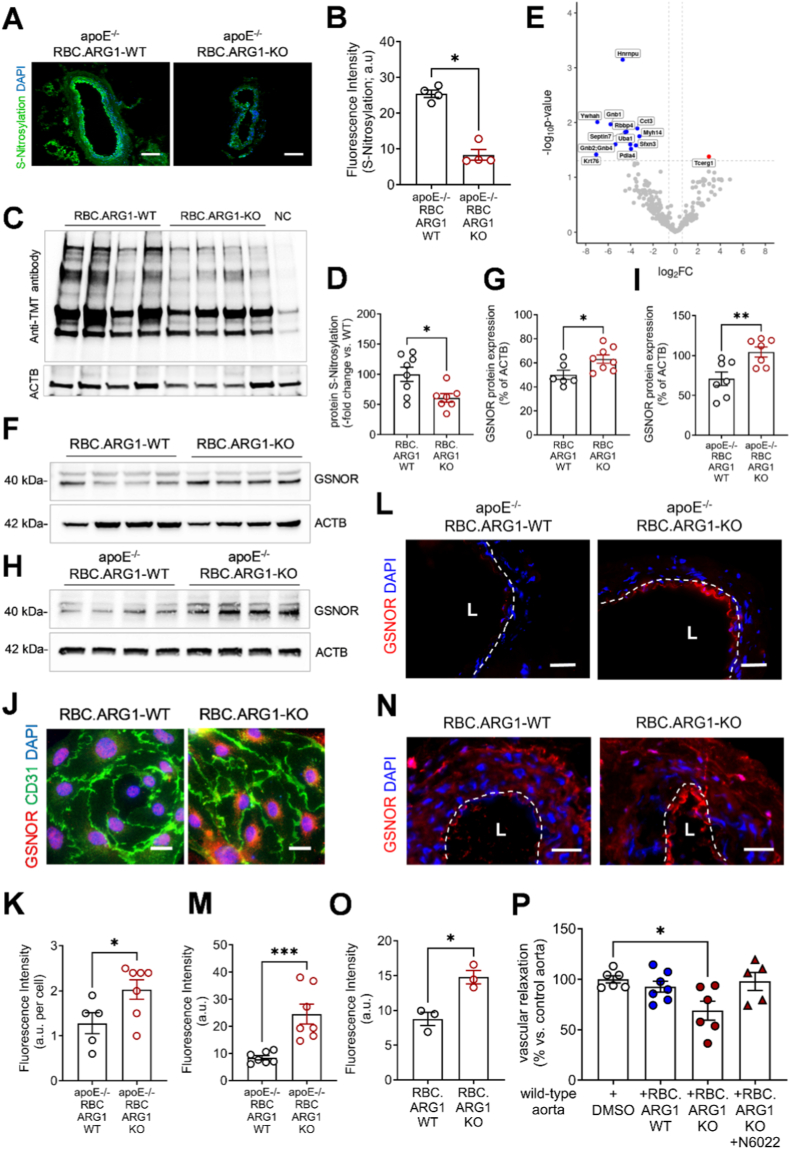
Endothelial cells from mice lacking ARG1 in erythrocytes exhibit endothelial protein S-denitrosylation and GSNOR inhibition rescues the impaired vascular response to acetylcholine. **A, B** Biotin switch analysis of aortic cross-sections from apoE^−/−^ RBC.ARG1-WT and apoE^−/−^RBC.ARG1-KO mice to detect protein S-nitrosation. Size bars represent 100 μm *P < 0.05 (Mann-Whitney test). **C, D** Immunoprecipitation of S-nitrosylated proteins from primary endothelial cells (ECs) isolated from C57BL/6 RBC.ARG1-WT and C57BL/6 RBC RBC.ARG1-KO mice. Representative immunoblot using anti-TMT antibodies detecting S-nitrosylated proteins and ACTB as loading control (C), and the results of the quantitative analysis (D; n = 7 biological replicates of primary ECs isolated from RBC.ARG1-KO mice and n = 8 RBC.ARG1-WT controls; NC is negative control). *P < 0.05 (unpaired Student's *t*-test). **E** Mass spectrometry identification of nitrosylated proteins in RBC.ARG1-KO ECs (n = 7 biological replicates of primary ECs isolated from C57BL/6 RBC.ARG1-KO mice and n = 5 RBC.ARG1-WT controls). Proteins were enriched for S-nitrosylation and quantified by label-free mass spectrometry. The x-axis shows the log2 fold change (log2FC) in nitrosylated protein abundance, calculated from log2-transformed Label-Free Quantification (LFQ) intensities (RBC.ARG1-KO vs. RBC.ARG1-WT). The y-axis shows the −log10 p-value derived from the Student's t-test comparing protein LFQ intensities between RBC.ARG1-KO and RBC.ARG1-WT groups. Each point represents one protein. Proteins passing the significance thresholds (p < 0.05 and log2FC) are highlighted and labeled in blue for downregulated (denitrosylated), red for upregulated (nitrosylated) and grey (unchanged among two groups). **F–I** Immunoblot analysis of GSNOR protein expression in primary endothelial cells isolated from C57BL/6 RBC.ARG1-WT and C57BL/6 RBC.ARG1-KO mice (F, G) or from apoE^−/−^ RBC.ARG1-WT and apoE^−/−^ RBC.ARG1-KO mice (H, I). Representative membranes (F, H) and quantitative analysis (G, I). *P < 0.05 and **P < 0.01 (unpaired Student's t-test). (n = 7-8 biological replicates. **J-O** Immunostaining and quantitative analysis of GSNOR expression in cultivated primary endothelial cells isolated from apoE^−/−^ RBC.ARG1-WT and apoE^−/−^ RBC.ARG1-KO mice (J, K), or in acetone-fixed sections through the aorta of apoE^−/−^ RBC.ARG1-WT and apoE^−/−^ RBC.ARG1-KO mice (L, M) or the mesenteric artery of C57BL/6 RBC.ARG1-WT and C57BL/6 RBC.ARG1-KO mice (N, O). Cell nuclei were stained using 4′ 6-diamidino-2-phenylindole (DAPI). Size bars in J represent 5 μm, size bars in L, N represent 20 μm. The artery lumen (L) is marked with a dotted line. **P** Vascular relaxation studies in aortic segments isolated from C57BL/6 wild-type mice (n = 6), co-incubated overnight with erythrocytes freshly isolated from C57BL/6 RBC.ARG1-WT or RBC.ARG1-KO mice, in the presence of 100 nM the GSNOR inhibitor N6022 or DMSO (vehicle control) alone. *P < 0.05 (one-way ANOVA, Sidak's multiple comparisons test).

***Endothelial cells from mice lacking ARG1 in erythrocytes exhibit endothelial protein S-denitrosylation and GSNOR inhibition rescues the impaired vascular response to acetylcholine.*** In addition to activating the cGMP signaling pathway, NO may also signal via its cellular reservoir S-Nitrosoglutathione (GSNO). We have previously shown that overexpression of S-nitrosoglutathione reductase (GSNOR) in primary SMCs from apoE^−/−^ RBC.ARG1-KO mice fed Western type diet for 20 weeks promotes their differentiation into osteoblast-like cells and vascular calcification [13]. Here, we wondered whether alterations in endothelial protein S-nitrosylation may impact vascular NO signaling. To study functional outcomes rather than structural changes of the vasculature, analyses were performed in mice 12 weeks-of-age fed standard rodent diet. Analyses of aortic cross sections using the biotin switch assay and fluorescence microscopy showed significantly reduced S-nitrosylation, including at the endothelial lining (Fig. 3A and B). TMT labeling of protein SNO-modifications confirmed that protein S-nitrosylation was significantly reduced in RBC.ARG1-KO ECs (Fig. 3C and D). Importantly, targeted redox proteomics confirmed lower S-nitrosylation of endothelial proteins and identified several candidates, including the guanine nucleotide-binding proteins G Protein Subunit Beta 1 (GNB1), GNB2 and GNB4, involved in heterotrimeric G protein signaling, Myosin Heavy Chain 14 (MYH14), a non-muscle myosin involved in calmodulin binding and cytoskeleton motor activity, and the transcriptional repressor heterogeneous nuclear ribonucleoprotein U (HNRPU) among the proteins exhibiting significantly reduced S-nitrosylation in ECs from RBC.ARG1-KO mice (Fig. 3E). Endothelial expression of *Adh5* (not shown) or GSNOR, the enzyme which catalyzes the breakdown of the intracellular NO reservoir S-nitrosoglutathione (GSNO) and thereby controls intracellular levels of GNSO and S-nitrosylated proteins [32], was significantly increased in RBC.ARG1-KO ECs isolated from mice on both the C57BL/6 (Fig. 3F and G) and the apoE^−/−^ (Fig. 3H and I) background explaining the observed lower protein S-nitrosylation. Increased endothelial GSNOR expression in mice lacking ARG1 in RBCs was further confirmed by immunofluorescence microscopy of primary ECs (Fig. 3J and K), as well as in cross sections through the aorta (Fig. 3L and M) and the mesenteric arteries (Fig. 3N and O). Importantly, the GSNOR inhibitor N6022 restored the impaired relaxation of wild-type aortic rings in the presence of RBC.ARG1-KO erythrocytes to levels seen in the presence of RBC.ARG1-WT erythrocytes or aortic rings incubated with equal volumes of vehicle (DMSO) alone and improved the impaired relaxation of aortic rings exposed to RBC.ARG1-KO erythrocytes in response to ACh (Fig. 3P).

Together, we conclude from our findings that erythrocyte ARG1 contributes to circulating NO levels and blood pressure regulation. On the other hand, chronically shifting the balance towards increased erythrocyte NO generation also impairs endothelium-dependent vascular functions via overactivated cGMP-dependent signaling pathways as well as endothelial GSNOR overexpression and denitrosylation of endothelial proteins, including adapter proteins in heterotrimeric G protein signaling.

## 4 Discussion

NO is a key mediator of endothelial functions and promotes SMC relaxation resulting in vasodilation to increase blood flow and to lower blood pressure levels. Numerous studies have documented the importance of eNOS for vascular homeostasis and the maintenance of a ‘healthy’, functional endothelium, whereas loss or dysfunction of eNOS is the starting point of cardiovascular diseases, including arterial hypertension and atherosclerosis. However, previous studies also have shown that not only diminished NO, but also chronically elevated NO levels or NO generated from other cellular sources promotes endothelial dysfunction [33]. For example, apoE^−/−^ mice with targeted endothelial overexpression of eNOS exhibited accelerated atherosclerosis progression, resulting from eNOS uncoupling and increased formation of NO-derived reactive oxygen species (ROS) [34,35]. Moreover, endothelial dysfunction was shown to coincide with enhanced eNOS expression and superoxide anion expression [36]. While our findings confirm the importance of NO for blood pressure regulation and highlight the contribution of RBCs therein, they also support the detrimental consequences of chronically increased NO production as a mediator of endothelial dysfunction. While a role for increased erythrocyte ROS production was ruled out in a previous study from our group [13], overactivated NO signaling via cGMP, but also the upregulation of counterregulatory mechanisms and overexpression of GSNOR was observed resulting in increased denitrosylation of endothelial proteins. We have previously shown that deletion of ARG1 in erythrocytes induces vascular calcification and increased aortic pulse pressure in the context of structural aortic remodeling after feeding apoE^−/−^ mice with western-type diet for twenty weeks [13]. In contrast, and to study functional outcomes rather than structural changes of their arteries, mice used in the present study were approximately 12 weeks-of-age and fed normal rodent diet. Our study is the first to examine changes in vascular NO signaling in response to altered NO release from RBCs and signifies an initial and mostly functional phase of the phenotype compared to the calcification-driven arterial stiffening which becomes more relevant at later stages and under Western type diet. Our observation of lower mean and diastolic blood pressure levels *in vivo* and impaired acetylcholine-induced vasorelaxation of isolated aortic rings or mesenteric arterioles *ex vivo* point towards complex vascular adaptations to the chronically increased circulating NO levels in RBC.ARG1-KO mice.

The production of NO by eNOS is counterregulated by ARG1 [5], and both enzymes compete for l-arginine, the only nitrogen source for the formation of NO [37]. Impaired NO production due to erythrocyte overexpression of ARG1 has been associated with endothelial vasodilation in mouse models and patients with metabolic risk factors, like obesity [27,38], diabetes [8,9,39] or hypercholesterolemia [8,9,40,41], and findings that systemic ARG1 inhibition [42,43] or knockdown [44] restored vascular function support a causal link. However, studies using cell-specific ARG1 are scarce. A recent study reports that plasma nitrite and blood pressure levels or vasorelaxation do not differ in mice with ARG1 deletion in cells expressing Cre recombinase under control of the hemoglobin b promoter [45], inducing gene deletion at the terminal stages of erythroid differentiation [46]. In our study, ARG1 was deleted in the progenitor stages of erythropoiesis, possibly explaining the presence of a vascular phenotype. Differences in genetic background (e.g. apoE^−/−^ vs. C57BL/6) also may have played a role, although vascular dysfunction was already seen in RBC.ARG1-KO mice in the present study and more pronounced in apoE^−/−^ RBC.ARG1-KO mice. In contrast to ARG1 deletion in RBCs, our data also suggest only a minor role for endothelial ARG1 for vascular function control, in line with previous reports in normoglycemic [47] as well as diabetic mice [27,28]. High expression of ARG1 expression in SMCs and preservation of total vascular ARG1 activity, as reported earlier [47], may have played a role.

The fact that no detectable phenotype of endothelial or erythrocyte ARG1 deletion was observed under basal conditions on a C57BL/6 background [45,47] makes an important point for the interpretation of our findings and suggests that arginase may have a cell type-specific as well as context-dependent roles [48]. Moreover, the marked species differences in ARG1 expression and activity in RBCs, which was recently found to be over 10,000 times lower in mice compared to humans [45,48], illustrate the limitations of directly extrapolating findings in murine models to human biology.

Binding of NO to the ferrous haem iron of soluble guanylate cyclase stimulates cGMP formation in SMCs resulting in vasodilation. In our study, increased NO signaling via cGMP in primary aortic SMCs from RBC.ARG1-KO mice was suggested by findings of increased VASP phosphorylation at the protein kinase G site serine-239. The increased cGMP levels could be the result of increased generation, for example from the intracellular NO receptor soluble GC, but also other receptors coupling to guanylyl cyclases involved in vascular function regulation, such as membrane-bound GCs activated by natriuretic peptides. A recent study suggested that sGC is activated by cGMP released from hypoxic RBCs [49]. GSNO is the precursor of S-nitroso-l-cysteine (Cys-NO), which also can activate sGC, as already shown in pulmonary artery SMCs [50]. In addition to NO signaling via cGMP, GMP-*in*dependent NO signaling pathways also exist and involve the reversible covalent adduction of NO to free thiol cysteine residues [51]. This so called protein S-nitrosylation may help to overcome the diffusible nature of NO and localize its effects [52], for example at the cell membrane or the sarcoplasmic reticulum [53]. S-nitrosylation was shown to stabilize dihydrofolate reductase, the enzyme involved in the regeneration of the eNOS cofactor tetrahydrobiopterin (BH4), and protect it from degradation thus preventing eNOS uncoupling [54]. However, targeted redox proteomics did not detect altered BH4 S-nitrosation in our study. Protein S-nitrosation of sGC resulting in impaired sGC activity has been suggested as a mechanism of sGC desensitization towards NO stimulation [55] and was found to occur in states of increased nitro-oxidative stress [56,57]. Our findings that aortic explants continue to respond to the NO donor glyceryl trinitrate with vasodilation do not support the presence of sGC desensitization in SMCs from RBC.ARG1-KO mice.

The equilibrium of protein S-nitrosylation is controlled by enzymes removing S-nitrosothiols from proteins, in particular GSNOR, which degrades the more stable storage form of NO, GSNO, to glutathione and NO [58]. GSNOR also functions as denitrosylase and removes nitrosothiols from cysteines in proteins. We have previously shown that overexpression of GSNOR in SMCs from apoE^−/−^ RBC.ARG1-KO mice promotes the denitrosylation of heat shock protein 70 and thereby contributes to vascular calcification [13]. GSNOR expression was also strongly increased in ECs from RBC.ARG1-KO mice. An overall reduced nitrosylation of endothelial proteins in RBC.ARG1-KO mice was suggested by the biotin switch technology and immunoblot analysis of S-nitrosocysteine protein modifications, and confirmed by targeted redox proteomic analysis. Regarding the significance of specific endothelial proteins showing reduced protein S-nitrosylation in our study, highly relevant candidates with regard to the observed phenotype were GNB1, GNB2 and GNB4, providing a possible explanation why the signaling via G-protein coupled receptors may be altered in those cells, including the response to acetylcholine. Changes in MYH14 S-nitrosylation may have affected cellular Ca^2+^ sensing by alternating its interaction with calmodulin [59]. We also identified reduced S-nitrosylation of the transcriptional repressor HNRPU, which could explain the observed changes in endothelial gene expression patterns. Previous work has shown that LPS-mediated increase in NO generation from macrophages alters the activity of HNRPU [60]. That these alterations lead to lower mean and diastolic blood pressure at the same time as impairing aortic vasorelaxation is surprising. Importantly, unbiased proteomics recently identified endothelial GSNOR overexpression as critical factor mediating the vascular complications in rodent models of diabetes as well as in patients [61]. GSNOR inhibition was found to improve vasodilation via mechanisms independent of its enzymatic activity via binding to ETS-related gene and triggering the nuclear export of CRM1 [61]. Of note, not all patients with endothelial dysfunction develop arterial hypertension. In patients with non-obstructive angina, endothelial dysfunction and an abnormal, paradoxial response to acetylcholine also can be observed, independent of the presence of hypertension [62]. Future studies will have to reveal the contribution of erythrocyte NO to this phenomenon.

In summary, our findings extend previous reports on the differential contribution of erythrocytes and endothelial cells to vascular function and blood pressure control. They particularly highlight the importance of erythrocyte ARG1 and its crosstalk with ECs, which may extend to biological pathways beyond the regulation of NO availability.

## Ethics declarations

The authors declare no financial or non-financial competing interests.

## Funding sources

This study was supported by research funding from the 10.13039/501100001659German Research Foundation (*Deutsche Forschungsgemeinschaft*; SCHA 808/9-2, project number 329795682, and SCHA 808/17-1, project number 555133650, to K.S.; and 10.13039/501100003383CRC 1531, project number 456687919, with A03 to 10.13039/100001818RPB, A09 to K.S., and S01 to I.W.), and the 10.13039/100010447German Center for Cardiovascular Research (Deutsches Zentrum für Herz-Kreislauf-Forschung, DZHK e.V.; cooperation with shared expertise, project number FKZ 81X2210131, to R.G., K.S, A.W. and M.F., and doctoral student fellowships to B.S.). P.W., R.P.B, M.F., P.L., and K.S. are Principal Investigators of the 10.13039/100010447DZHK, R.G., M.L.B., I.W., F.R., and A.W. are 10.13039/100010447DZHK scientists, B.S., M.L., P.G. and M.M. are members of the Young-10.13039/100010447DZHK.

## CRediT authorship contribution statement

**Rajinikanth Gogiraju:** Conceptualization, Formal analysis, Funding acquisition, Investigation, Methodology, Writing – review & editing. **Beichen Sun:** Formal analysis, Investigation. **Magdalena L. Bochenek:** Formal analysis, Investigation, Writing – review & editing. **Ilka Wittig:** Formal analysis, Funding acquisition, Methodology. **Flávia Rezende:** Funding acquisition, Methodology. **Melina Lopez:** Investigation. **Angela Wirth:** Investigation, Methodology. **Wenjia Zhao:** Methodology. **Elsa W. Böhm:** Methodology. **Payal Guliani:** Formal analysis, Investigation. **Kateryna Moiko:** Formal analysis, Investigation. **Michael Molitor:** Formal analysis, Investigation, Methodology. **Philip Wenzel:** Methodology, Resources. **Marc Freichel:** Funding acquisition, Resources, Supervision, Writing – review & editing. **Philipp Lurz:** Resources. **Ralf P. Brandes:** Funding acquisition, Resources, Supervision, Writing – review & editing. **Katrin Schäfer:** Conceptualization, Funding acquisition, Project administration, Supervision, Writing – original draft.

## Declaration of competing interest

The authors declare that they have no known competing financial interests or personal relationships that could have appeared to influence the work reported in this paper.

## Data Availability

Data will be made available on request.

## References

[1] Farah C., Michel L.Y.M., Balligand J.L. (2018). Nitric oxide signalling in cardiovascular health and disease. Nat. Rev. Cardiol..

[2] Kleinbongard P., Schulz R., Rassaf T., Lauer T., Dejam A., Jax T., Kumara I., Gharini P., Kabanova S., Ozuyaman B., Schnurch H.G., Godecke A., Weber A.A., Robenek M., Robenek H., Bloch W., Rosen P., Kelm M. (2006). Red blood cells express a functional endothelial nitric oxide synthase. Blood.

[3] Wood K.C., Cortese-Krott M.M., Kovacic J.C., Noguchi A., Liu V.B., Wang X., Raghavachari N., Boehm M., Kato G.J., Kelm M., Gladwin M.T. (2013). Circulating blood endothelial nitric oxide synthase contributes to the regulation of systemic blood pressure and nitrite homeostasis. Arterioscler. Thromb. Vasc. Biol..

[4] Leo F., Suvorava T., Heuser S.K., Li J., LoBue A., Barbarino F., Piragine E., Schneckmann R., Hutzler B., Good M.E., Fernandez B.O., Vornholz L., Rogers S., Doctor A., Grandoch M., Stegbauer J., Weitzberg E., Feelisch M., Lundberg J.O., Isakson B.E., Kelm M., Cortese-Krott M.M. (2021). Red blood cell and endothelial eNOS independently regulate circulating nitric oxide metabolites and blood pressure. Circulation.

[5] Yang J., Gonon A.T., Sjoquist P.O., Lundberg J.O., Pernow J. (2013). Arginase regulates red blood cell nitric oxide synthase and export of cardioprotective nitric oxide bioactivity. Proc. Natl. Acad. Sci. U. S. A.

[6] Durante W., Johnson F.K., Johnson R.A. (2007). Arginase: a critical regulator of nitric oxide synthesis and vascular function. Clin. Exp. Pharmacol. Physiol..

[7] Kim P.S., Iyer R.K., Lu K.V., Yu H., Karimi A., Kern R.M., Tai D.K., Cederbaum S.D., Grody W.W. (2002). Expression of the liver form of arginase in erythrocytes. Mol. Genet. Metabol..

[8] Zhou Z., Mahdi A., Tratsiakovich Y., Zahoran S., Kovamees O., Nordin F., Uribe Gonzalez A.E., Alvarsson M., Ostenson C.G., Andersson D.C., Hedin U., Hermesz E., Lundberg J.O., Yang J., Pernow J. (2018). Erythrocytes from patients with type 2 diabetes induce endothelial dysfunction via arginase I. J. Am. Coll. Cardiol..

[9] Yang J., Zheng X., Mahdi A., Zhou Z., Tratsiakovich Y., Jiao T., Kiss A., Kovamees O., Alvarsson M., Catrina S.B., Lundberg J.O., Brismar K., Pernow J. (2018). Red blood cells in type 2 diabetes impair cardiac post-ischemic recovery through an arginase-dependent modulation of nitric oxide synthase and reactive oxygen species. JACC, Basic Transl. Sci..

[10] Mahdi A., Wodaje T., Kövamees O., Tengbom J., Zhao A., Jiao T., Henricsson M., Yang J., Zhou Z., Nieminen A.I., Levin M., Collado A., Brinck J., Pernow J. (2023). The red blood cell as a mediator of endothelial dysfunction in patients with familial hypercholesterolemia and dyslipidemia. J. Intern. Med..

[11] Heinrich A.C., Pelanda R., Klingmuller U. (2004). A mouse model for visualization and conditional mutations in the erythroid lineage. Blood.

[12] Forde A., Constien R., Gröne H.-J., Hämmerling G., Arnold B. (2002). Temporal Cre-mediated recombination exclusively in endothelial cells using Tie2 regulatory elements. Genesis.

[13] Gogiraju R., Renner L., Bochenek M.L., Zifkos K., Molitor M., Danckwardt S., Wenzel P., Münzel T., Konstantinides S., Schäfer K. (2022). Arginase-1 deletion in erythrocytes promotes vascular calcification via enhanced GSNOR (S-Nitrosoglutathione reductase) expression and NO signaling in smooth muscle cells. Arterioscler. Thromb. Vasc. Biol..

[14] Percie du Sert N., Hurst V., Ahluwalia A., Alam S., Avey M.T., Baker M., Browne W.J., Clark A., Cuthill I.C., Dirnagl U., Emerson M., Garner P., Holgate S.T., Howells D.W., Karp N.A., Lazic S.E., Lidster K., MacCallum C.J., Macleod M., Pearl E.J., Petersen O.H., Rawle F., Reynolds P., Rooney K., Sena E.S., Silberberg S.D., Steckler T., Würbel H. (2020). The ARRIVE guidelines 2.0: updated guidelines for reporting animal research. PLoS Biol..

[15] Lopez M., Malacarne P.F., Gajos-Draus A., Ding X., Daiber A., Lundberg J.O., Offermanns S., Brandes R.P., Rezende F. (2021). Vascular biotransformation of organic nitrates is independent of cytochrome P450 monooxygenases. Br. J. Pharmacol..

[16] Zifkos K., Bochenek M.L., Gogiraju R., Robert S., Pedrosa D., Kiouptsi K., Moiko K., Wagner M., Mahfoud F., Poncelet P., Münzel T., Ruf W., Reinhardt C., Panicot-Dubois L., Dubois C., Schäfer K. (2024). Endothelial PTP1B deletion promotes VWF exocytosis and venous thromboinflammation. Circ. Res..

[17] Jin Y., Liu Y., Antonyak M., Peng X. (2012). Isolation and characterization of vascular endothelial cells from murine heart and lung. Methods Mol. Biol..

[18] Wong E., Nguyen N., Hellman J. (2021). Isolation of primary mouse lung endothelial cells. J. Vis. Exp..

[19] Ghasemi I., Gogiraju R., Khraisat S., Pagel S., Graf C., Brandt M., Madhusudhan T., Wenzel P., Luxán G., Lurz P., Bochenek M.L., Schäfer K. (2025). Side-by-Side comparison of culture media uncovers phenotypic and functional differences in primary mouse aortic mural cells. Cells.

[20] Wirth A., Benyó Z., Lukasova M., Leutgeb B., Wettschureck N., Gorbey S., Örsy P., Horváth B., Maser-Gluth C., Greiner E., Lemmer B., Schütz G., Gutkind J.S., Offermanns S. (2008). G12-G13–LARG–mediated signaling in vascular smooth muscle is required for salt-induced hypertension. Nat. Med..

[21] Wenzel P., Knorr M., Kossmann S., Stratmann J., Hausding M., Schuhmacher S., Karbach S.H., Schwenk M., Yogev N., Schulz E., Oelze M., Grabbe S., Jonuleit H., Becker C., Daiber A., Waisman A., Münzel T. (2011). Lysozyme M–Positive monocytes mediate angiotensin II–Induced arterial hypertension and vascular dysfunction. Circulation.

[22] Zhuge Z., McCann Haworth S., Nihlén C., Carvalho L., Heuser S.K., Kleschyov A.L., Nasiell J., Cortese-Krott M.M., Weitzberg E., Lundberg J.O., Carlström M. (2023). Red blood cells from endothelial nitric oxide synthase-deficient mice induce vascular dysfunction involving oxidative stress and endothelial arginase I. Redox Biol..

[23] Green L.S., Chun L.E., Patton A.K., Sun X., Rosenthal G.J., Richards J.P. (2012). Mechanism of inhibition for N6022, a first-in-class drug targeting S-Nitrosoglutathione reductase. Biochemistry.

[24] Frenis K., Helmstädter J., Ruan Y., Schramm E., Kalinovic S., Kröller-Schön S., Bayo Jimenez M.T., Hahad O., Oelze M., Jiang S., Wenzel P., Sommer C.J., Frauenknecht K.B.M., Waisman A., Gericke A., Daiber A., Münzel T., Steven S. (2021). Ablation of lysozyme M-positive cells prevents aircraft noise-induced vascular damage without improving cerebral side effects. Basic Res. Cardiol..

[25] Löwe O., Rezende F., Heidler J., Wittig I., Helfinger V., Brandes R.P., Schröder K. (2019). BIAM switch assay coupled to mass spectrometry identifies novel redox targets of NADPH oxidase 4. Redox Biol..

[26] Ettehad D., Emdin C.A., Kiran A., Anderson S.G., Callender T., Emberson J., Chalmers J., Rodgers A., Rahimi K. (2016). Blood pressure lowering for prevention of cardiovascular disease and death: a systematic review and meta-analysis. Lancet.

[27] Bhatta A., Yao L., Xu Z., Toque H.A., Chen J., Atawia R.T., Fouda A.Y., Bagi Z., Lucas R., Caldwell R.B., Caldwell R.W. (2017). Obesity-induced vascular dysfunction and arterial stiffening requires endothelial cell arginase 1. Cardiovasc. Res..

[28] Chennupati R., Meens M.J., Janssen B.J., van Dijk P., Hakvoort T.B.M., Lamers W.H., De Mey J.G.R., Koehler S.E. (2018). Deletion of endothelial arginase 1 does not improve vasomotor function in diabetic mice. Physiol. Rep..

[29] Ma J., Li Y., Yang X., Liu K., Zhang X., Zuo X., Ye R., Wang Z., Shi R., Meng Q., Chen X. (2023). Signaling pathways in vascular function and hypertension: molecular mechanisms and therapeutic interventions. Signal Transduct. Targeted Ther..

[30] Mo E., Amin H., Bianco I.H., Garthwaite J. (2004). Kinetics of a cellular nitric oxide/cGMP/phosphodiesterase-5 pathway. J. Biol. Chem..

[31] Held K.F., Dostmann W.R. (2012). Sub-nanomolar sensitivity of nitric oxide mediated regulation of cGMP and vasomotor reactivity in vascular smooth muscle. Front. Pharmacol..

[32] Liu L., Hausladen A., Zeng M., Que L., Heitman J., Stamler J.S. (2001). A metabolic enzyme for S-nitrosothiol conserved from bacteria to humans. Nature.

[33] Wever R.M., Lüscher T.F., Cosentino F., Rabelink T.J. (1998). Atherosclerosis and the two faces of endothelial nitric oxide synthase. Circulation.

[34] Ozaki M., Kawashima S., Yamashita T., Hirase T., Namiki M., Inoue N., Hirata K., Yasui H., Sakurai H., Yoshida Y., Masada M., Yokoyama M. (2002). Overexpression of endothelial nitric oxide synthase accelerates atherosclerotic lesion formation in apoE-deficient mice. J. Clin. Investig..

[35] Takaya T., Hirata K., Yamashita T., Shinohara M., Sasaki N., Inoue N., Yada T., Goto M., Fukatsu A., Hayashi T., Alp N.J., Channon K.M., Yokoyama M., Kawashima S. (2007). A specific role for eNOS-derived reactive oxygen species in atherosclerosis progression. Arterioscler. Thromb. Vasc. Biol..

[36] Bouloumie A., Bauersachs J., Linz W., Scholkens B.A., Wiemer G., Fleming I., Busse R. (1997). Endothelial dysfunction coincides with an enhanced nitric oxide synthase expression and superoxide anion production. Hypertension.

[37] Palmer R.M., Ashton D.S., Moncada S. (1988). Vascular endothelial cells synthesize nitric oxide from L-arginine. Nature.

[38] Johnson F.K., Peyton K.J., Liu X.M., Azam M.A., Shebib A.R., Johnson R.A., Durante W. (2015). Arginase promotes endothelial dysfunction and hypertension in Obese rats. Obesity.

[39] Beleznai T., Feher A., Spielvogel D., Lansman S.L., Bagi Z. (2011). Arginase 1 contributes to diminished coronary arteriolar dilation in patients with diabetes. Am. J. Physiol. Heart Circ. Physiol..

[40] Wang W., Hein T.W., Zhang C., Zawieja D.C., Liao J.C., Kuo L. (2011). Oxidized low-density lipoprotein inhibits nitric oxide-mediated coronary arteriolar dilation by up-regulating endothelial arginase I. Microcirculation.

[41] Kovamees O., Shemyakin A., Eriksson M., Angelin B., Pernow J. (2016). Arginase inhibition improves endothelial function in patients with familial hypercholesterolaemia irrespective of their cholesterol levels. J. Intern. Med..

[42] Johnson F.K., Johnson R.A., Peyton K.J., Durante W. (2005). Arginase inhibition restores arteriolar endothelial function in Dahl rats with salt-induced hypertension. Am. J. Physiol. Regul. Integr. Comp. Physiol..

[43] Mahdi A., Kovamees O., Checa A., Wheelock C.E., von Heijne M., Alvarsson M., Pernow J. (2018). Arginase inhibition improves endothelial function in patients with type 2 diabetes mellitus despite intensive glucose-lowering therapy. J. Intern. Med..

[44] White A.R., Ryoo S., Li D., Champion H.C., Steppan J., Wang D., Nyhan D., Shoukas A.A., Hare J.M., Berkowitz D.E. (2006). Knockdown of arginase I restores NO signaling in the vasculature of old rats. Hypertension.

[45] Heuser S.K., Li J., Li Z., LoBue A., Heard K., Hocks J., Suvorava T., Cadeddu R.P., Strupp C., Dunaway L., Zhuge Z., Gelhaus S.L., Heinen A., Germing U., Feelisch M., Carlström M., Isakson B., Kelm M., Lundberg J.O., Cortese-Krott M.M. (2025). Divergent roles of red cell arginase in humans and mice: RBC Arg1 KO mice show preserved systemic l-arginine bioavailability and infarct size in vivo. Redox Biol..

[46] Elliott S., Sinclair A.M. (2012). The effect of erythropoietin on normal and neoplastic cells. Biologics.

[47] Heuser S.K., LoBue A., Li J., Zhuge Z., Leo F., Suvorava T., Olsson A., Schneckmann R., Guimaraes Braga D.D., Srivrastava T., Montero L., Schmitz O.J., Schmitt J.P., Grandoch M., Weitzberg E., Lundberg J.O., Pernow J., Kelm M., Carlström M., Cortese-Krott M.M. (2022). Downregulation of eNOS and preserved endothelial function in endothelial-specific arginase 1-deficient mice. Nitric Oxide : Biol. Chem..

[48] Heuser S.K., Li J., Pudewell S., LoBue A., Li Z., Cortese-Krott M.M. (2025). Biochemistry, pharmacology, and in vivo function of arginases. Pharmacol. Rev..

[49] Yang J., Sundqvist M.L., Zheng X., Jiao T., Collado A., Tratsiakovich Y., Mahdi A., Tengbom J., Mergia E., Catrina S.B., Zhou Z., Carlström M., Akaike T., Cortese-Krott M.M., Weitzberg E., Lundberg J.O., Pernow J. (2023). Hypoxic erythrocytes mediate cardioprotection through activation of soluble guanylate cyclase and release of cyclic GMP. J. Clin. Investig..

[50] Riego J.A., Broniowska K.A., Kettenhofen N.J., Hogg N. (2009). Activation and inhibition of soluble guanylyl cyclase by S-nitrosocysteine: involvement of amino acid transport system L. Free Radic. Biol. Med..

[51] Stamler J.S., Simon D.I., Osborne J.A., Mullins M.E., Jaraki O., Michel T., Singel D.J., Loscalzo J. (1992). S-nitrosylation of proteins with nitric oxide: synthesis and characterization of biologically active compounds. Proc. Natl. Acad. Sci. U. S. A.

[52] Iwakiri Y., Satoh A., Chatterjee S., Toomre D.K., Chalouni C.M., Fulton D., Groszmann R.J., Shah V.H., Sessa W.C. (2006). Nitric oxide synthase generates nitric oxide locally to regulate compartmentalized protein S-nitrosylation and protein trafficking. Proc. Natl. Acad. Sci. U. S. A.

[53] Barouch L.A., Harrison R.W., Skaf M.W., Rosas G.O., Cappola T.P., Kobeissi Z.A., Hobai I.A., Lemmon C.A., Burnett A.L., O'Rourke B., Rodriguez E.R., Huang P.L., Lima J.A., Berkowitz D.E., Hare J.M. (2002). Nitric oxide regulates the heart by spatial confinement of nitric oxide synthase isoforms. Nature.

[54] Cai Z., Lu Q., Ding Y., Wang Q., Xiao L., Song P., Zou M.H. (2015). Endothelial nitric oxide synthase-derived nitric oxide prevents dihydrofolate reductase degradation via promoting S-Nitrosylation. Arterioscler. Thromb. Vasc. Biol..

[55] Sayed N., Baskaran P., Ma X., van den Akker F., Beuve A. (2007). Desensitization of soluble guanylyl cyclase, the NO receptor, by S-nitrosylation. Proc. Natl. Acad. Sci. U. S. A.

[56] Crassous P.A., Couloubaly S., Huang C., Zhou Z., Baskaran P., Kim D.D., Papapetropoulos A., Fioramonti X., Durán W.N., Beuve A. (2012). Soluble guanylyl cyclase is a target of angiotensin II-induced nitrosative stress in a hypertensive rat model. Am. J. Physiol. Heart Circ. Physiol..

[57] Kossmann S., Hu H., Steven S., Schonfelder T., Fraccarollo D., Mikhed Y., Brahler M., Knorr M., Brandt M., Karbach S.H., Becker C., Oelze M., Bauersachs J., Widder J., Munzel T., Daiber A., Wenzel P. (2014). Inflammatory monocytes determine endothelial nitric-oxide synthase uncoupling and nitro-oxidative stress induced by angiotensin II. J. Biol. Chem..

[58] Whalen E.J., Foster M.W., Matsumoto A., Ozawa K., Violin J.D., Que L.G., Nelson C.D., Benhar M., Keys J.R., Rockman H.A., Koch W.J., Daaka Y., Lefkowitz R.J., Stamler J.S. (2007). Regulation of beta-adrenergic receptor signaling by S-nitrosylation of G-protein-coupled receptor kinase 2. Cell.

[59] Stencel M.G., VerMeer M., Giles J., Tran Q.K. (2022). Endothelial regulation of calmodulin expression and eNOS-calmodulin interaction in vascular smooth muscle. Mol. Cell. Biochem..

[60] Gao C., Guo H., Wei J., Mi Z., Wai P., Kuo P.C. (2004). S-Nitrosylation of heterogeneous nuclear ribonucleoprotein A/B regulates osteopontin transcription in endotoxin-stimulated murine macrophages. J. Biol. Chem..

[61] Zhao S., Song T., Tang X., Fan C., Yang Y., Zhang Z., Xia Y., Zhang Y., Cao J., Wang Z., Shi Z., Tang X., Wang D., Yin G., Zhang S., Gao Y., Chen H., Wang L., Chen F., Wang H., Yu B., Cao Y., Sun K., Liu X., Wang X., Yan C., Han Y., Han Y., Xie L., Ji Y. (2025). *S*-nitrosoglutathione reductase as a therapeutic target for diabetic vascular complications in rodent models. Sci. Transl. Med..

[62] Miner S., Mejia-Renteria H., Leone A.M., Velollari O., Sykes R., Biscaglia S., Esposito G., Galante D., Oreglia J., Ang D., Weferling M., Di Serafino L., Berry C., Campo G., Escaned J., Crea F., Gori T. (2025). Endotypes of angina with non-obstructive coronary arteries: a prospective multicentre study. Eur. Heart J..

